# Conserved signatures of the canine faecal microbiome are associated with metronidazole treatment and recovery

**DOI:** 10.1038/s41598-024-51338-7

**Published:** 2024-03-04

**Authors:** Zoe V. Marshall-Jones, Krusha V. Patel, Juan Castillo-Fernandez, Zoe N. Lonsdale, Richard Haydock, Ruth Staunton, Gregory C. A. Amos, Phillip Watson

**Affiliations:** Waltham Petcare Science Institute, Waltham on the Wolds, Leicestershire LE14 4RT UK

**Keywords:** Microbiology, Dysbiosis

## Abstract

Antibiotic resistance is recognised as one of the biggest global threats to human and animal health. Understanding the influence of antibiotics on the canine microbiome is important to know the potential mid-to-long term effects on dysbiosis and mitigate side-effects such as antibiotic-associated diarrhoea. In this study, metronidazole was prescribed to 22 dogs for suspected giardiasis after exhibiting gastrointestinal symptoms such as diarrhoea and/or vomiting. Faecal samples were collected before, during seven days of treatment, and six months post-cessation. Faecal microbiota was assessed with 16S rRNA sequencing. Shannon diversity was reduced for up to three days after the treatment ended, and an altered community persisted for four to six weeks. All dogs recovered to a similar microbiome composition as pre-treatment. Immediately after receiving metronidazole, an increase in the relative abundance of the genera *Lactobacillus*, *Bifidobacterium*, and *Enterococcus* was observed. This may be due to antibiotic resistance commonly exhibited by these organisms. One-to-two weeks post-cessation, several other genera that were sensitive to the antibiotic recovered in abundances, with taxa belonging to the Erysipelotrichaceae family particularly driving composition change. Many of the bacteria initially reduced were associated with carbohydrate fermentation. This suggests scope exists to explore interventions to augment gastrointestinal health and support the re-establishment of the microbiome.

## Introduction

The compositional and functional characteristics of the gastrointestinal microbiota play a vital role in the overall health and nutritional status of humans and animals^1^. Extensive research into the gut microbiome of humans and mice have resulted in links being drawn between microbial community composition and local gastrointestinal health and resilience in disorders such as Crohn’s disease, ulcerative colitis, and irritable bowel disorder^2–4^. Systemic conditions as diverse as allergies, arthritis, weight maintenance, and diabetes mellitus as well as host behaviours such as anxiety and aggression have also been associated with gastrointestinal microbiome signatures^5–8^.

As found in humans, despite key differences in nutritional intake, the gastrointestinal microbiota of cats and dogs represents a highly complex ecosystem consisting of several hundred bacterial taxa^9–11^. In line with the co-evolution of the gut microbiota in mammals, molecular surveys describe Firmicutes, Bacteroidetes, Proteobacteria, Fusobacteria, and Actinobacteria as representing the most abundant phyla in the gut and faecal samples of pets. At higher levels of taxonomic resolution, the individual nature of the microbiome within individuals is well recognised, as is its influence on health and the physiological response to nutrition and medicines^12^. Following the initial colonisation of the gut and development of diversity during maturation, a dynamic community unique to the individual host fluctuates in composition around a stable average state in the absence of dietary change or perturbation^13–15^. Local gastrointestinal and oral conditions have also been reported to have wider impacts on the microbiome and therefore pet health^16–22^.

As with human medicine, there is a strategic focus to reduce antibiotic administration in veterinary medicine and policies in small animal practices, however there is still a need for broad-spectrum antibiotics^23^. As such, it is important to understand the impact of antibiotics on the microbiome in pets. Reduced diversity and altered microbial community structure have been described both during and following antibiotic treatment in both humans and a range of animals, in some cases over prolonged periods^24–27^. For example, exploring the effect of amoxicillin on the faecal microbiota of dogs revealed reductions in richness and diversity, albeit short-lived, with a recovery to baseline after treatment cessation^28^. A seven-day prescription of tylosin to a cohort of 16 healthy adult dogs resulted in a shift in the faecal microbiome, with a decrease in bacterial diversity, specifically *Fusobacteriaceae* and *Veillonellaceae*, and a continued altered abundance of some species eight weeks after cessation, with at least two months recommended to restore the native microbiome^29^. A study in five dogs to assess the impact of metronidazole treatment resulted an altered bacterial composition at the family level, with resolution reported within 28 days of treatment cessation^30^. Metronidazole administration when explored in combination with different diet types also reported significant changes to the microbial communities, with decreases in observed taxa in richness as well as Shannon index, that remained unresolved after four weeks^31^.

The assessment of antibiotic usage must be conducted in a responsible manner, utilising clinical populations to avoid inappropriate use of antibiotics which may be detrimental to the control and management of antibiotic resistance^32^. This has been discussed in a recent review on metronidazole treatment in dogs^33^. Another consideration is the highly individual nature of the faecal microbiota and its variability over time. This presents challenges in cross-sectional and population-based studies due to confounders within cohorts, particularly with smaller groups^18,34,35^. Finally, the health status of the study participants is important. Antibiotics will be primarily prescribed to manage disease, therefore studies on healthy animals may not reflect the impact of antibiotics on the populations they are most likely be prescribed to treat.

We conducted a time-course study to explore the effect that metronidazole has on the microbiome of dogs when prescribed for a suspected Giardia infection and provide a better understanding of the subsequent recovery of the bacterial community following treatment cessation. Faecal parameters were also taken to provide the context of clinical signs of gastrointestinal health.

## Results

### Diversity of the faecal microbiome changes with metronidazole treatment

Changes to the gut microbiome were determined across a 25-week period spanning before, during and after metronidazole treatment via faeces collection and targeted sequencing of the V4 region of the 16S rRNA gene. From 22 dogs, a total of 241 faeces samples were profiled, which resulted in the identification of 1,131 ASVs.

**Figure 1:**
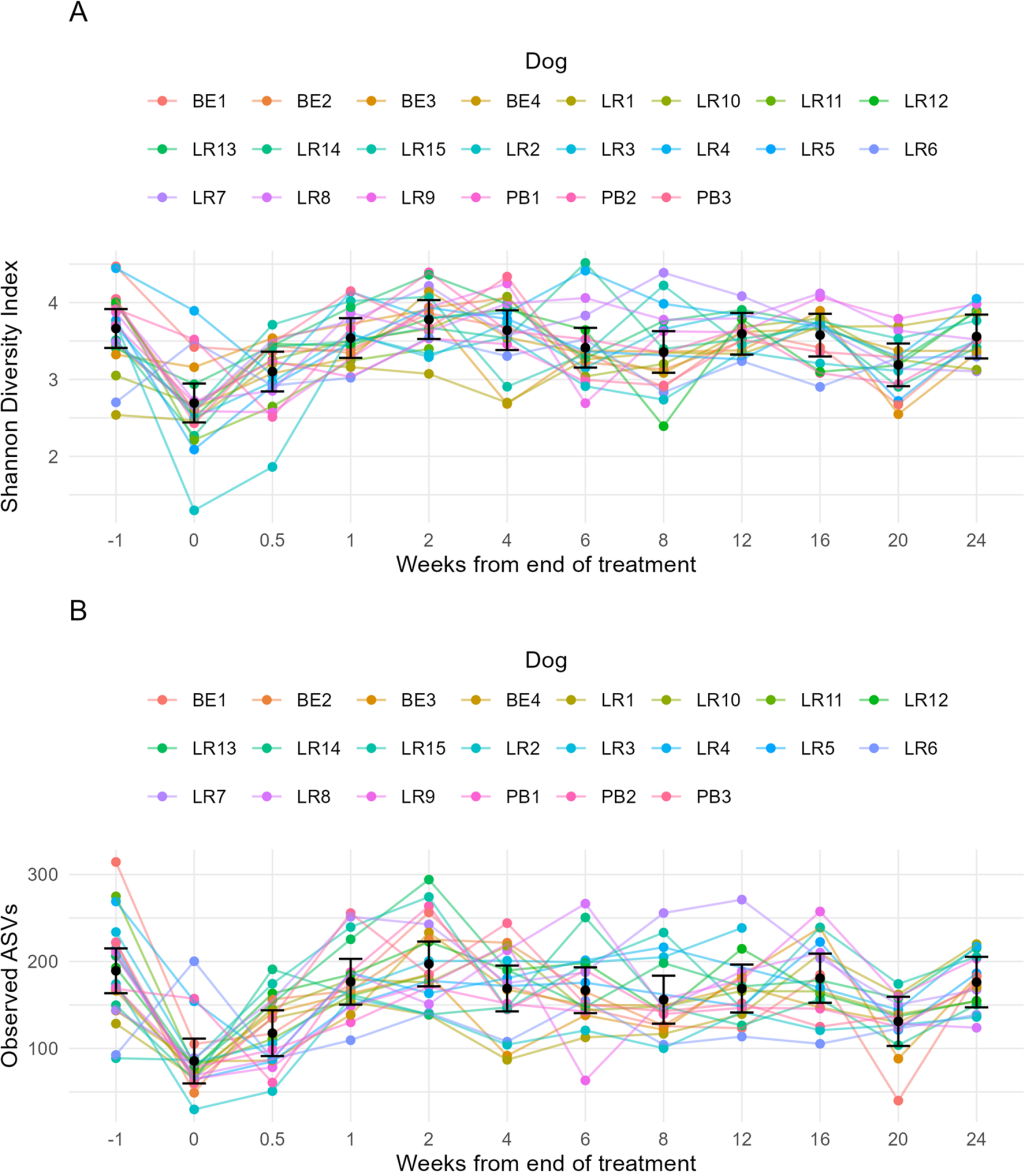
Longitudinal effect for individual dogs [Week−1 (prior to treatment), Week 0 (treatment), Week 1 onwards (treatment cessation)]. Error bars represent 95% confidence intervals. (**A**)**.** For Shannon diversity index scores. (**B**). For species richness represented as observed ASVs.

Bacterial alpha diversity was estimated using Shannon diversity index scores which accounts for both abundance and evenness of the species present within a sample (Fig. 1A; Tables 1, 2). Prior to antibiotic administration, the mean diversity was 3.66 (95% CI (3.41, 3.92)); this fell to 2.69 (95% CI (2.44, 2.95)) after antibiotic treatment (Week 0) for all dogs across the cohort (mean difference, − 0.97; 95% CI (− 1.29, − 0.65); *p* < 0.001). Three days later, alpha diversity increased to 3.10 (95% CI (2.84, 3.36)). However, it was still significantly reduced compared to pre-treatment (mean difference − 0.56; 95% CI (− 0.88, − 0.24); *p* < 0.001). Resolution of the clinical signs were observed by the veterinarian from Week 1. Comparatively, there was a distinction in the magnitude of mean differences between the differences at Week 1 and 2 (0.12 and 0.12, respectively) to the comparably larger 0.56 and 0.97 found in the weeks prior. Finally, no significant differences in diversity compared to pre-treatment were observed for the remainder of the study, except for Week 20 (mean difference − 0.47; 95% CI (− 0.81, − 0.13); *p* = 0.001); however, this fluctuation was smaller than that observed at either Week 0 or Week 0.5.

**Table 1:** Shannon diversity index with estimated means (95% confidence intervals) over time [Week− 1 (prior to treatment), Week 0 (treatment), Week 1 onwards (treatment cessation)].

Week	Estimated mean	95% lower	95% upper
− 1	3.66	3.41	3.92
0	2.69	2.44	2.95
0.5	3.10	2.84	3.36
1	3.54	3.28	3.80
2	3.78	3.53	4.03
4	3.64	3.38	3.90
6	3.41	3.15	3.67
8	3.36	3.09	3.63
12	3.59	3.32	3.86
16	3.58	3.30	3.85
20	3.19	2.91	3.47
24	3.56	3.27	3.84

**Table 2:** Shannon diversity indices comparing mean differences (95% confidence intervals) between Week− 1 (prior to treatment) and all other timepoints [Week 0 (treatment), Week 1 onwards (treatment cessation)].

Comparison	Mean difference	95% lower	95% upper	*P* value
0 versus − 1	− 0.97	− 1.29	− 0.65	<0.001*
0.5 versus − 1	− 0.56	− 0.88	− 0.24	<0.001*
1 versus − 1	− 0.12	− 0.45	0.20	0.93
2 versus − 1	0.12	− 0.20	0.44	0.95
4 versus − 1	− 0.02	− 0.35	0.30	1.00
6 versus − 1	− 0.25	− 0.57	0.07	0.23
8 versus − 1	− 0.30	− 0.64	0.03	0.09
12 versus − 1	− 0.07	− 0.40	0.26	1.00
16 versus − 1	− 0.09	− 0.42	0.25	1.00
20 versus − 1	− 0.47	− 0.81	− 0.13	0.001*
24 versus − 1	− 0.10	− 0.45	0.24	0.98

*P* values below 0.05 are considered statistically significant—denoted with an asterisk (*).

Observed ASVs (species richness) was also explored (Fig. 1B; Tables S2, S3). The greatest significant difference was again observed between Week−1 (prior to treatment) and Week 0 (treatment) (mean difference − 103.74; 95% CI (− 136.37, − 71.11); *p* < 0.001). As above, slight fluctuations with the estimated mean numbers of observed ASVs was noted following Week 4 onwards, but these were relatively stable. The exception to this again was at Week 20 post treatment, when compared to Week−1 (mean difference − 58.14; 95% CI (− 92.66, − 23.62); *p* < 0.001), a significant difference in the estimated means was found.

**Figure 2:**
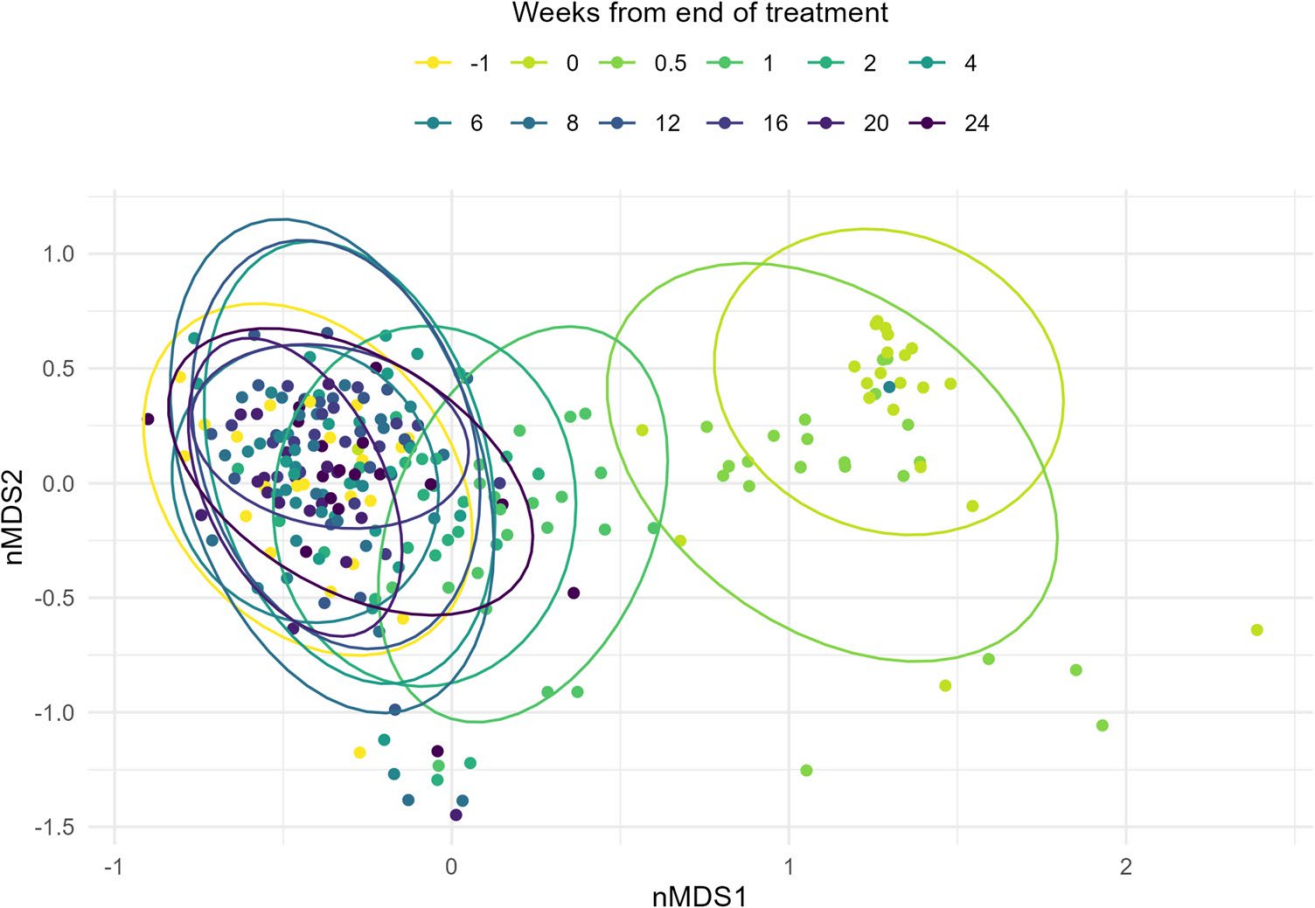
Bray-Curtis (nMDS) beta diversity plot to represent the ASVs from all individuals over time [Week−1 (prior to treatment), Week 0 (treatment), Week 1 onwards (treatment cessation)]. Ellipses represent 95% confidence intervals.

Beta diversity was estimated using Bray-Curtis to visually assess dissimilarity between samples (Fig. 2). This showed a clear distinction from the samples collected before treatment (Week−1) to those from the treatment phase (Week 0) across the nMDS1 axis. An overlap in communities was seen between the treatment and immediately post-treatment (Week 0.5). With each subsequent collection, there was a further shift from the treatment clusters towards pre-treatment (Week−1), with samples from Week 6 onwards showing strong similarity with overlapping ellipses. These observations concurred with pair-wise comparisons with PERMANOVA and Bonferroni adjustments (Table 3). The strongest R^2^ values were 0.46 [Weeks−1 and 0 (adjusted *p* value = 0.01)] and 0.42 [Weeks−1 and 0.5 (adjusted *p* value = 0.01)]. Contrastingly, when comparing Week 6 following cessation to Week−1 an R^2^ value of 0.04 (adjusted *p* value = 0.06) was obtained.

**Table 3:** Pair-wise comparisons for Week−1 (prior to antibiotic treatment) and all other timepoints [Week 0 (treatment), Week 1 onwards (treatment cessation)] using PERMANOVA.

Comparison	R^2^	*P* value	Adjusted *P* value
0 versus − 1	0.46	0.001	0.01*
0.5 versus − 1	0.42	0.001	0.01*
1 versus − 1	0.20	0.001	0.01*
2 versus − 1	0.13	0.001	0.01*
4 versus − 1	0.07	0.001	0.01*
6 versus − 1	0.04	0.005	0.06
8 versus − 1	0.04	0.010	0.11
12 versus − 1	0.05	0.049	0.54
16 versus − 1	0.05	0.027	0.30
20 versus − 1	0.06	0.002	0.02*
24 versus − 1	0.03	0.169	1.00

*P* values below 0.05 are considered statistically significant—denoted with an asterisk (*)—after Bonferroni adjustment of significance levels.

### Faecal microbiome composition during metronidazole treatment and recovery

**Figure 3:**
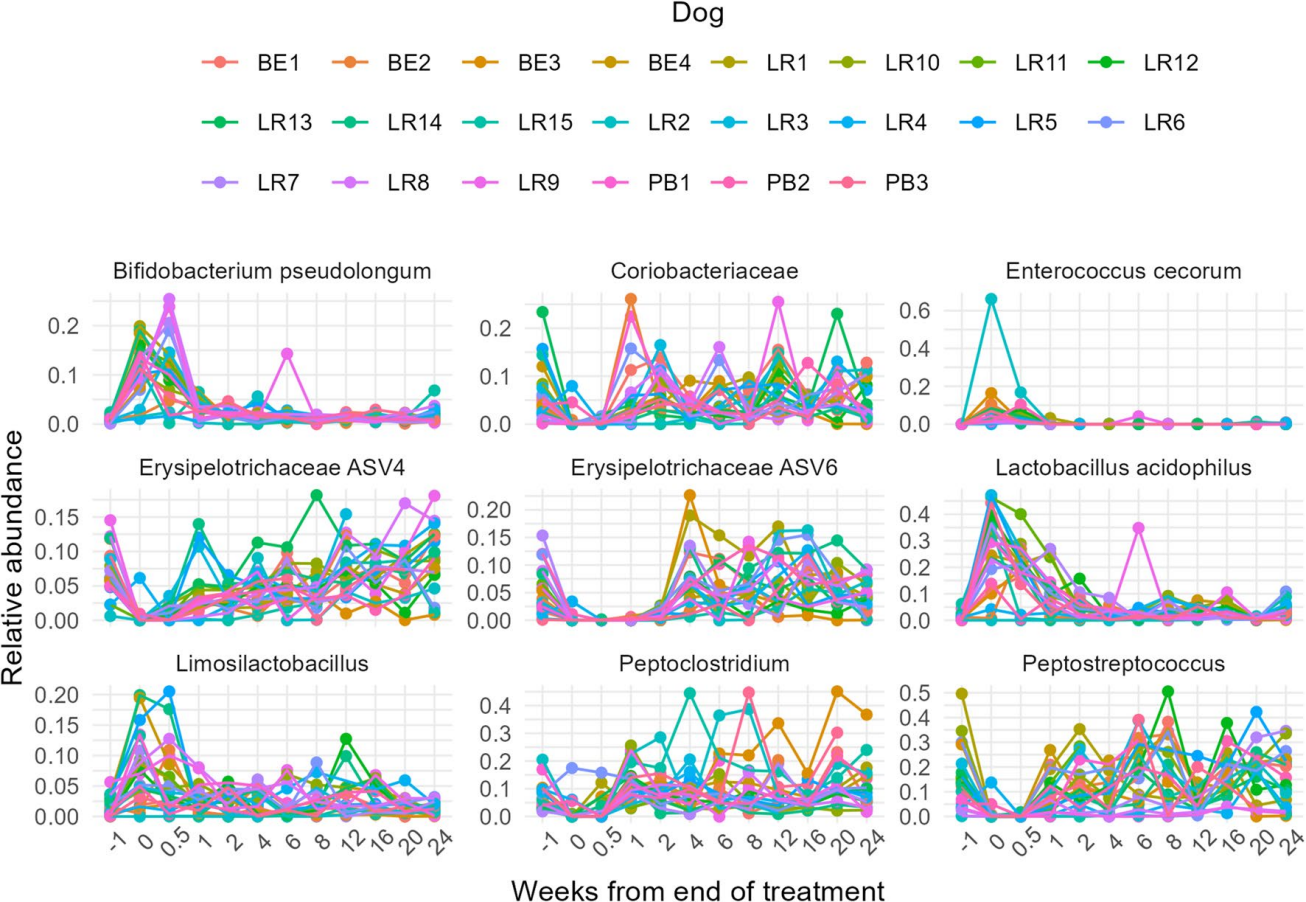
Relative abundance plots to represent the top 9 of 27 key ASVs from all individuals over time [Week−1 (prior to treatment), Week 0 (treatment), Week 1 onwards (treatment cessation)].

To further investigate shifts in the microbiota due to metronidazole treatment, the ASVs predominantly contributing to the Bray-Curtis dissimilarity between Week−1 and Week 0 were explored (Fig. 3, Table 4). A total of 27 ASVs were found to contribute to 70% of the observed dissimilarity. These clustered within six families: Lactobacillaceae, Peptostreptococcaceae, Bifidobacteriaceae, Enterococcaceae, Erysipelotrichaceae, and Coriobacteriaceae, with only ASV19 and ASV23 unable to be classified. With some ASVs, an increase in relative abundance was observed between Weeks 0 and 0.5 followed by a sharp decline at Week 1 (following antibiotic cessation). Examples include *Bifidobacterium longum*, *Enterococcus cecorum*, *Limosilactobacillus*, and *Lactobacillus acidophilus*; interestingly, these are all lactic acid bacteria. Inversely, one of the six Erysipelotrichaceae taxa identified—ASV6—had a different response in that the relative abundance declined immediately with the administration of metronidazole, remaining close to zero and only following two weeks from the end of the treatment, was a rapid proliferation in the number of taxa observed. For some taxa, such as Erysipelotrichaceae ASV4, *Peptoclostridium* and *Peptostreptococcus*, a decline in the relative abundance was observed during the antibiotic phase, but this was restored within a week of treatment cessation.

**Table 4:** Contribution of 27 most prevalent species responsible for driving Bray-Curtis dissimilarity between Week−1 (prior to treatment) and Week 0 (antibiotic treatment).

ASV	Taxa	Average Week−1	Average Week 0	Cumulative contribution	*P* value
ASV3	Bacteria; Firmicutes; Bacilli; Lactobacillales; Lactobacillaceae; Lactobacillus; acidophilus	0.01	0.27	0.15	0.001*
ASV1	Bacteria; Firmicutes; Clostridia; Peptostreptococcales-Tissierellales; Peptostreptococcaceae; Peptostreptococcus	0.15	0.01	0.23	0.001*
ASV9	Bacteria; Actinobacteriota; Actinobacteria; Bifidobacteriales; Bifidobacteriaceae; Bifidobacterium; pseudolongum	0.01	0.11	0.29	0.001*
ASV2	Bacteria; Firmicutes; Clostridia; Peptostreptococcales-Tissierellales; Peptostreptococcaceae; Peptoclostridium	0.08	0.01	0.33	0.001*
ASV25	Bacteria; Firmicutes; Bacilli; Lactobacillales; Enterococcaceae; Enterococcus; cecorum	<0.01	0.07	0.37	0.001*
ASV4	Bacteria; Firmicutes; Bacilli; Erysipelotrichales; Erysipelotrichaceae	0.07	0.00	0.41	0.001*
ASV8	Bacteria; Firmicutes; Bacilli; Lactobacillales; Lactobacillaceae; *Limosilactobacillus*	0.02	0.08	0.45	0.001*
ASV5	Bacteria; Actinobacteriota; Coriobacteriia; Coriobacteriales; Atopobiaceae; Coriobacteriaceae	0.06	0.01	0.48	0.001*
ASV6	Bacteria; Firmicutes; Bacilli; Erysipelotrichales; Erysipelotrichaceae	0.05	0.00	0.51	0.001*
ASV7	Bacteria; Firmicutes; Bacilli; Erysipelotrichales; Erysipelotrichaceae	0.04	0.00	0.53	0.001*
ASV12	Bacteria; Firmicutes; Bacilli; Lactobacillales; Lactobacillaceae; Lactobacillus; johnsonii	<0.01	0.04	0.55	0.001*
ASV10	Bacteria; Firmicutes; Bacilli; Erysipelotrichales; Erysipelotrichaceae	0.03	0.00	0.57	0.001*
ASV70	Bacteria; Actinobacteriota; Actinobacteria; Bifidobacteriales; Bifidobacteriaceae; Bifidobacterium; pseudocatenulatum	<0.01	0.02	0.58	0.001*
ASV31	Bacteria; Actinobacteriota; Actinobacteria; Bifidobacteriales; Bifidobacteriaceae; Bifidobacterium; pseudolongum	<0.01	0.02	0.59	0.001*
ASV28	Bacteria; Proteobacteria; Gammaproteobacteria; Enterobacterales; Enterobacteriaceae; Escherichia-Shigella; coli	<0.01	0.02	0.60	0.001*
ASV17	Bacteria; Firmicutes; Clostridia; Peptostreptococcales-Tissierellales; Peptostreptococcaceae; Romboutsia	0.02	0.00	0.61	0.001*
ASV38	Bacteria; Firmicutes; Bacilli; Erysipelotrichales; Erysipelotrichaceae; Dubosiella	<0.01	0.02	0.62	0.001*
ASV72	Bacteria; Firmicutes; Clostridia; Peptostreptococcales-Tissierellales; Peptostreptococcaceae; Terrisporobacter	0.00	0.02	0.63	0.001*
ASV36	Bacteria; Firmicutes; Bacilli; Lactobacillales; Enterococcaceae; Enterococcus; faecium	0.00	0.01	0.64	0.001*
ASV11	Bacteria; Firmicutes; Bacilli; Erysipelotrichales; Erysipelotrichaceae; Allobaculum	0.01	0.00	0.65	0.001*
ASV19	NA	0.01	0.02	0.66	0.166
ASV23	NA	0.01	0.00	0.66	0.001*
ASV22	Bacteria; Actinobacteriota; Actinobacteria; Bifidobacteriales; Bifidobacteriaceae; Bifidobacterium; animalis	0.01	0.02	0.67	0.107
ASV67	Bacteria; Actinobacteriota; Actinobacteria; Bifidobacteriales; Bifidobacteriaceae; Bifidobacterium; pullorum	<0.01	0.01	0.68	0.001*
ASV32	Bacteria; Firmicutes; Bacilli; Lactobacillales; Lactobacillaceae; *Limosilactobacillus*	<0.01	0.01	0.69	0.001*
ASV88	Bacteria; Firmicutes; Bacilli; Lactobacillales; Enterococcaceae; Enterococcus; cecorum	0.00	0.01	0.69	0.001*
ASV90	Bacteria; Firmicutes; Bacilli; Lactobacillales; Enterococcaceae; Enterococcus; cecorum	0.00	0.01	0.70	0.001*

*P* values below 0.05 are considered statistically significant—denoted with an asterisk (*).

### Faeces quality in dogs changes during metronidazole treatment and recovery

**Figure 4:**
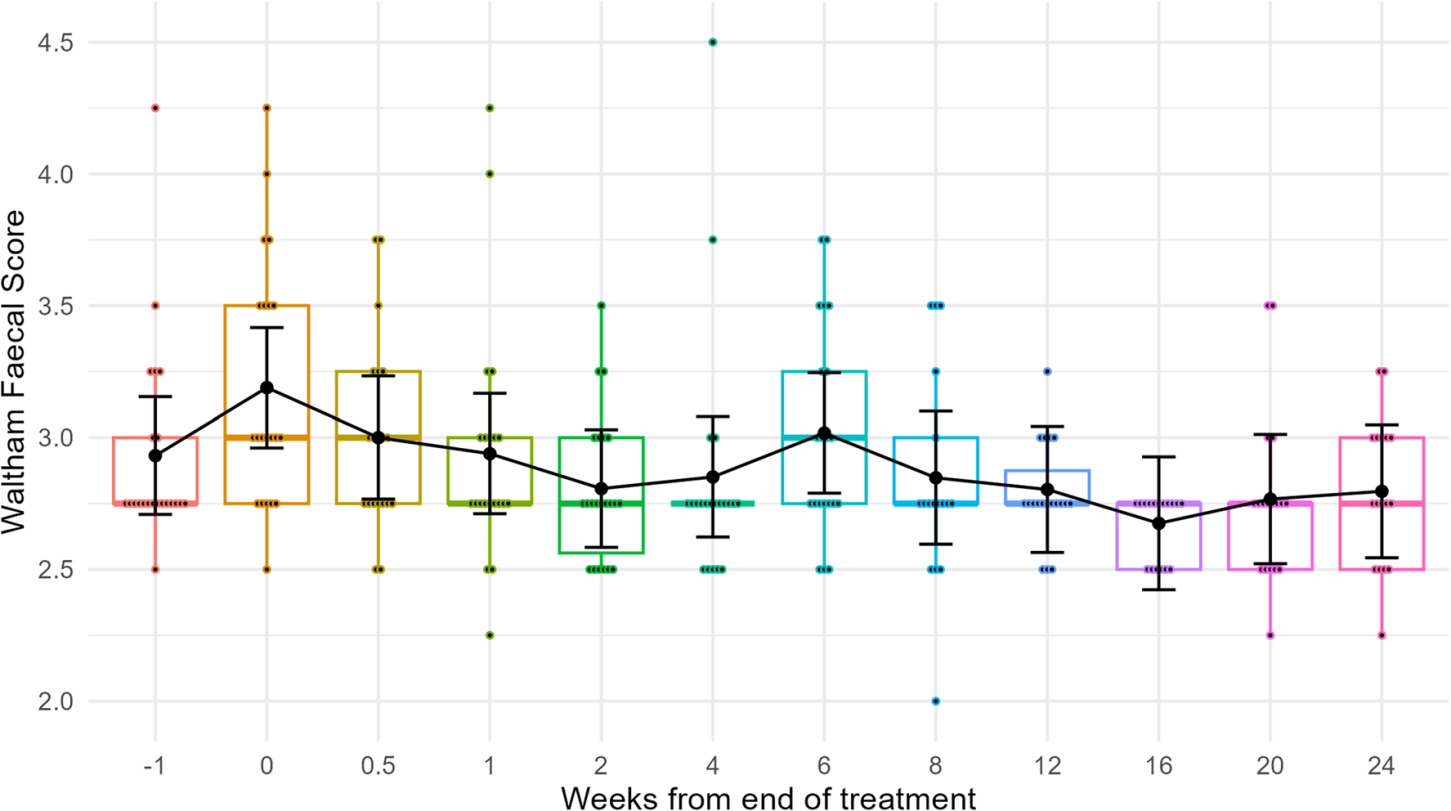
Box plots to represent the average faecal scores for all individuals over time [Week−1 (prior to treatment), Week 0 (treatment), Week 1 onwards (treatment cessation)]. Overlaid black line denotes the average for each timepoint. Error bars represent 95% confidence intervals. Optimal canine faeces consistency is 2.50.

Faeces quality was scored for dogs co-housed in pairs before and continuing for over 24 weeks after metronidazole treatment (Fig. 4). Variation in faeces scores were observed over time with a mean score that peaked at 3.19 (95% CI (2.96, 3.42)) at the time of the end of antibiotic treatment followed by a gradual stabilisation around 2.75, closer to the optimal score of 2.50^36^. A small increase in mean faeces score from all dogs was detected from Week−1 (prior to administration of antibiotics) to Week 0 (mean difference 0.26; 95% CI (− 0.03, 0.55); *p* = 0.12), which is not enough to confidently conclude that metronidazole treatment influences faeces consistency (Table S4).

## Discussion

This research describes the impact of metronidazole on the canine faecal microbiota of 22 dogs undergoing treatment for suspected giardiasis. Samples were collected before, during and for six months after antibiotic treatment. Gastrointestinal symptoms such as diarrhoea and/or vomiting were resolved following the seven-day course of metronidazole and animals recovered rapidly, as assessed by the attending veterinarian.

Changes to the microbial community with antibiotics have been explored in previous studies. Gentamycin treatment in children reportedly has long-term effects; a reduced diversity in faecal *Bifidobacterium* spp., lowered abundance of lactobacilli and some Actinobacteria taxa, and an increased abundance of Proteobacteria were reported, compared to untreated controls after eight weeks^25^. In a long-term microbiota prospective study with ciprofloxacin, structural changes were observed to vary between human adults as well as on repeat treatments. This suggests the exact effects may be influenced by the initial microbial population. Microbiota stabilisation was observed by ten months after treatment, although this community structure was different when compared to the pre-treatment microbiota within individuals^24^.

In this current study, a significant reduction in diversity was observed with metronidazole administration (up to three days after end of treatment), with microbiota recovery occurring after only one week post treatment cessation. Diversity was highly variable both between individuals and over time; this variability is an accepted feature of the gut microbiota of individuals. An example was in Week 20, when fluctuations in species diversity and richness were observed; we hypothesise these changes could be attributed to natural perturbations to the canine microbiota. In line with the observed reduction in diversity, compositional changes in the faecal microbiota occurred most dramatically within the first week of treatment, and slowly recovered to the original composition over the following four to six weeks. These findings concur with metronidazole administration in cohorts of healthy dogs such that significant changes to the microbial species richness and community have been reported, with these either not being resolved at the point of study completion at four weeks, or persisting for between four to eight weeks^30,31,37^.

The microbial composition during and immediately following treatment was dominated by lactic acid bacteria (*Bifidobacterium* and *Enterococcus*), as well as two Lactobacillaceae genera (*Lactobacillus* and *Limosilactobacillus*). Their consistent presence in samples collected immediately after treatment across the cohort as well as being key taxa responsible for driving the beta diversity, suggest these ASVs may represent a core microbiota naturally resistant to metronidazole. Indeed, studies prior have demonstrated the resistance of certain *Lactobacillus, Bifidobacterium,* and *Enterococcus*^38–40^*.* The increased relative abundance of these bacteria during dysbiosis suggests an important role in microbiome re-establishment for these genera, with all reported to be involved in short chain fatty acid production and bacteriocins that have a range of health benefits including immune modulation and colonisation resistance.

In the one to two weeks following the completion of antibiotics, a change in the genera represented was apparent with sequence types assigned to *Allobaculum, Peptoclostridium*, and *Peptostreptococcus* spp. increasing in abundance as well as ASVs assigned to *Blautia* and an unclassified Erysipelotrichaceae genus returning to represent abundant phylotypes in the canine faecal microbiota. This suggests a sensitivity to the antibiotics, with growth occurring post-cessation after an initial drop in abundance. Indeed, analysis of the ASVs responsible for driving composition changes identified six taxa from the Erysipelotrichaceae family. Of these, five were unclassified by genera, but one was confirmed as *Allobaculum*. These characteristics of re-establishment of the gut microbiota following metronidazole treatment point to the importance of microbes involved in the metabolism of complex carbohydrates in the canine gut, consistent with the omnivorous evolutionary niche of dogs. This suggests that there is scope to further explore interventions to augment gastrointestinal health and support the re-establishment of the microbiota. Members of the Erysipelotrichaceae family have been associated with influencing metabolic parameters associated with immunity, obesity and insulin resistance and are enriched by prebiotic fibre^41^. Differences in the mode of action of antibiotics, and consequently in the sensitivity of microbes to antibiotics, mean that the findings of this study should be considered in the context of the specific antibiotic used with the relevant dose and time course involved.

Faecal quality was assessed throughout the study using the 17-point Waltham Faeces Scoring System^33^. The mean faecal score stabilised from a peak of 3.19, as recorded at the end of the seven-day metronidazole treatment, to around 2.75, in line with the return to the pre-antibiotic level of bacterial diversity. This is in line with previous research associating the faecal microbiome with the faecal scores^42^.

We recognise a limitation of this study is neither the faecal quality nor microbiota of the individuals was known prior to the development of suspected giardiasis. We also do not know whether the return to baseline represents recovery to the pre-disease state. This is because dogs were only recruited to this study following the attending veterinarian’s decision to prescribe antibiotics, and so the first sample being collected after the cohort were exhibiting gastrointestinal symptoms. This differs from other studies exploring the effect of metronidazole whereby healthy dogs have been recruited^29,31,37^. However, this may mean the results from our opportunistic study will more closely represent the microbial changes and time to recovery experienced by client-owned dogs.

Supportive therapy aiming to reduce the likelihood of antibiotic-associated diarrhoea is becoming commonplace in veterinary practice and is often effective for the individual in the immediate term. As such, probiotic species are increasingly prescribed alongside antibiotics^43^. The findings reported here indicate a potential risk in the use of probiotics with inherently plastic genomes and proactive gene transfer mechanisms such as *Enterococcus* species^32,44–47^. Caution should therefore be exercised with regards to the supportive therapy administered, and with the use of probiotic preparations. Concurrent therapy providing both a recipient bacterial species and antibiotic selective pressure may represent a risk in the development of antibiotic resistance given the predominance of naturally occurring enterococci in the gastrointestinal tract of dogs receiving metronidazole treatment. Furthermore, the findings that common probiotic species increase in relative abundance post-metronidazole exposure questions the requirement to further supplement with these species.

## Conclusion

Taken together, these findings suggest that the use of metronidazole in dogs is associated with an enhanced relative abundance of lactic acid bacteria and a reduction in diversity as metronidazole sensitive taxa are cleared. Recovery of gut microbiota diversity was observed following a significant decrease in the Shannon diversity and restoration of the microbiota composition, within four to six weeks following the cessation of antibiotics. Based on these findings, opportunities may exist for interventions to enhance gastrointestinal health during the re-establishment of the microbiota following treatment with metronidazole through the promotion of health-associated microbial taxa.

## Materials and methods

### Animal cohort

A cohort of 22 dogs at the Waltham Petcare Science Institute (Leicestershire, UK) were recruited to an opportunistic study following prescription of a seven-day course of metronidazole treatment at a daily dose of 25 mg/kg; this ranged from 400 to 500 mg per dog. The treatment was prescribed for veterinary management of a suspected Giardia infection, after gastrointestinal signs were observed including mucoid bloody diarrhoea and sporadic vomiting. Dogs were pair-housed in environmentally-enriched kennel facilities^48^. Coprophagia management was not exercised and therefore is a limitation of the study. This study was approved by the Waltham Animal Welfare and Ethical Review Body (80265) and conducted under the authority of the Animals (Scientific Procedures) Act 1986.

### Study design

Faeces consistency assessments were conducted daily according to the 17-point Waltham Faeces Scoring System^36^. Freshly produced defecations were collected from individual dogs for assessment of the faecal microbiota in the 24 h following prescription but prior to administration, during the seven-day administration, and at regular intervals in the period up to six months following antibiotic treatment.

The cohort comprised 8 neutered males and 14 females (12 neutered and 2 entire), aged between 0.6 and 8.8 years, from three breeds: Petit Basset Griffon Vendéens, Labrador retrievers and Beagles. The dogs were involved in their normal daily activities and were exercised consistently throughout the study according to standard practice for the institute. Although diet was not controlled throughout the six-month period, most dogs received the same commercially available nutritionally complete dry kibble dog food. Animals received their standard diet allocation as determined for the individual dog to provide energy levels required for bodyweight and body condition score maintenance. Dogs were always provided with access to fresh drinking water.

To elucidate the cause of the poor faecal quality (diarrhoea) and vomiting, faecal samples were submitted to a reference laboratory for microbiological analysis with the subsequent root cause being determined as giardiasis. Animals were assessed throughout the treatment and recovery by the attending veterinarian.

### Sample and data collections

A total of 12 faeces samples comprising of the first defecation of the day were collected from each individual dog with first immediately prior to antibiotic treatment and subsequent collections after six or seven days of receiving metronidazole and following the completion of the prescribed seven-day antibiotic treatment, as described below. Faeces collection time points spanned two days to enable freshly evacuated defecations to be obtained for the entire cohort. Post-antibiotic treatment collections were conducted at the start (two to three days post-antibiotic treatment) and end of the first week after the completion of treatment (seven to eight days post-antibiotic treatment), again at two, four, six, and eight weeks, and monthly up to six months after the cessation of antibiotics. Metadata collected included animal age, sex, bodyweight, breed, collection time point, and diet.

During the study, five dogs from the cohort met the exclusion criteria due to repeat antibiotic or probiotic/prebiotic (Promax, Vetplus UK) administration following veterinary advice. Repeated measures collected for excluded individuals were included in the final study dataset until their exclusion point (Table S1). The earliest exclusion was at time point 6, four weeks after the completion of metronidazole treatment. Exclusions from the final dataset included: Dog LR11 excluded from sampling occasion 6; Dog PB1 excluded from sampling occasion 8; Dog BE2 and Dog LR3 excluded from sampling occasion 10 and Dog PB3 excluded from sampling occasion 12. Additionally, microbiota measures were missed at one faeces collection time point for four dogs (LR4, LR5, LR13 and BE3). All non-missing observations were used in faecal microbiota analyses.

### Faecal microbiota sample preparation

Evacuated faeces representing the first defecation of the day were collected from individuals immediately prior to or immediately following feeding. A 200 mg sample was obtained from the core of the faeces using sterile disposable spatulas (Fisher Scientific, UK) no longer than 15 min after defecation. Samples were stored in Lo-Bind Eppendorf tubes (Eppendorf Ltd., UK) at − 80 °C within 60 min of production.

Faecal DNA was extracted using the QIAamp 96 PowerFecal QIAcube HT Kit (QIAGEN, Germany) with automation of extraction protocols on an epMotion 5075 robot (Eppendorf Ltd., UK). Briefly, suspended faecal material was homogenised by bead beating on a TissueLyser (QIAGEN, Germany) for 5 min at 30 Hz, and treatment with proteinase K (Qiagen) for 10 min (all at room temperature). Faecal lysates were applied to the QIAamp 96 well extraction plate under a 900-mbar vacuum prior to washing and elution according to the manufacturer’s instructions. Purified DNA was analysed by Nanodrop spectrophotometry and quantified with a Qubit 2.0 fluorometer (Invitrogen Europe Ltd., UK), according to manufacturer’s instructions.

Amplification of the 16S rRNA gene from extracted faecal DNA was conducted by PCR using dual-indexed primers targeting the V4 region (319F/806R primer pairs) according to Fadrosh et al.^49^. Amplifications were conducted from 10 ng of faecal DNA using Phusion High-Fidelity polymerase (New England Biolabs) according to manufacturer’s instructions with 0.1 µM primer preparations. Initial denaturation of the DNA target was conducted at 98 °C for 30 s followed by 30 cycles of sequential denaturation, annealing and elongation according to the following protocol: 98 °C for 15 s, 58 °C for 15 s, 72 °C for 15 s and a final extension of 60 s at 72 °C. Amplification controls included DNA negative, DNA extraction negatives, and positive controls including a 40-strain genomic DNA mix and mixed faecal preparation at 10 ng/µl. Visualization of the amplicons following separation by agarose gel electrophoresis compared to the 1 Kb ladder (Invitrogen) was used to confirm amplification of the target region. Sequence analysis of the resulting V4 16S rRNA gene amplicons was conducted using the Illumina MiSeq platform (Eurofins Genomics, Germany), according to standard protocols. All samples, regardless of timepoint, were randomised across two plates.

### Sequencing and bioinformatic analyses of faecal bacterial community composition

Assessments of DNA sequence data quality were made through data summaries describing the average and range of sequence reads per sample. Sequencing read thresholds of a minimum 1000 sequence reads per sample were used to ensure consistency in DNA sequence data across the samples. Sequence reads from forward and reverse primers were assembled into contiguous sequences representing the V3-V4 region of the 16S rRNA gene using FLASH assembler^50^. Dual indexing barcodes were removed using TagCleaner^51^. Next, demultiplexing of sequence reads was conducted in QIIME1 using split_libraries_fastq.py. DADA2 was used to infer amplicon sequence variants (ASVs) and remove chimeric sequences^52^. ASVs were retained for analysis if they had a relative abundance > 0.01% in at least two samples. Taxonomic assignment was conducted by searches against the SILVA database release 138^53^ using blast 2.13.0 at a sequence identity ≥ 97%^54^.

### Analysis of diversity in the faecal microbiota

Alpha diversity was assessed by Shannon diversity and species richness on rarefied counts to the minimum sample size (*n* = 2947) selected at random. This was repeated 50 times and the mean Shannon and species richness was calculated for each sample. Alpha diversity was modelled using a linear mixed model with time point included as a fixed effect and dog as a random effect to account for the repeat measurements within each dog. Dunnett’s multiple comparison test was used to assess alpha diversity differences before metronidazole administration and subsequent timepoints. All analyses were performed in R (version 4.1.3) using the vegan, phyloseq, lme4, and multcomp libraries^55^.

### Analysis of faecal microbial community composition

Beta diversity was assessed by Bray-Curtis dissimilarity on relative abundances of filtered ASVs (relative abundance > 0.01% in at least two samples). Differences between timepoints were assessed using pairwise permutational analysis of variance (PERMANOVA) with 999 permutations and constrained by stratification within individual dogs. *P* values were adjusted using Bonferroni correction. The individual contribution of amplicon sequence variants (ASVs) to the Bray-Curtis dissimilarity between samples taken before metronidazole administration and those collected at the end of the seven-day treatment were estimated using the similarity percentage (simper) method with 999 permutations. All analyses were performed in R (version 4.1.3) using the vegan and pairwise Adonis libraries^55^.

### Analysis of faeces consistency

Faeces consistency was assessed by trained animal husbandry technicians according to the 17-point Waltham Faeces Scoring System^36^. Acceptable faeces scores are between 1.50 and 3.75^56^. Faecal scores were analysed by linear mixed model using time point as a fixed effect and dog as a random effect to account for repeated measures. Dunnett’s multiple comparison test was used to assess faecal score differences in samples collected before metronidazole administration and subsequent timepoints. All analyses were performed in R using the lme4 and multcomp libraries^55^.

### Ethics approval and consent for publication

This study was approved by the Waltham Animal Welfare and Ethical Review Body (80265) and conducted under the authority of the Animals (Scientific Procedures) Act 1986. The authors complied with the ARRIVE guidelines. All methods were performed in accordance with the relevant guidelines and regulations.

### Supplementary Information

Supplementary Video 1.

Supplementary Information.

Supplementary Table 1.

Supplementary Table 2.

Supplementary Table 3.

Supplementary Table 4.

## Data Availability

The datasets generated during and/or analysed during the current study are available from the corresponding author on reasonable request.
