# Supplementary material for: Conserved signatures of the canine faecal microbiome are associated with metronidazole treatment and recovery
Source: Sci Rep. 2024 Mar 4;14:5277. doi: 10.1038/s41598-024-51338-7 (PMC10912219; doi:10.1038/s41598-024-51338-7)
Supplement: Supplementary file 2 — Supplementary Information. [file 41598_2024_51338_MOESM2_ESM.docx]

### Supplementary Figure 1. Interactive Bray-Curtis (nMDS) beta diversity plot to represent the ASVs from all individuals over time [Week -1 (prior to treatment), Week 0 (treatment), Week 1 onwards (treatment cessation)]. Ellipses represent 95% confidence intervals.
