# Supplementary material for: Conserved signatures of the canine faecal microbiome are associated with metronidazole treatment and recovery
Source: Sci Rep. 2024 Mar 4;14:5277. doi: 10.1038/s41598-024-51338-7 (PMC10912219; doi:10.1038/s41598-024-51338-7)
Supplement: Supplementary file 4 — Supplementary Table 2. [file 41598_2024_51338_MOESM4_ESM.docx]

### Table S2. Species Richness represented as observed ASVs with estimated means (95% confidence intervals) over time [Week -1 (prior to treatment), Week 0 (treatment), Week 1 onwards (treatment cessation)].

| Week | Estimated mean | 95% lower | 95% upper |
| --- | --- | --- | --- |
| -1 | 189.29 | 163.52 | 215.06 |
| 0 | 85.55 | 59.79 | 111.32 |
| 0.5 | 117.57 | 91.26 | 143.89 |
| 1 | 176.69 | 150.38 | 203.01 |
| 2 | 197.15 | 171.39 | 222.92 |
| 4 | 168.95 | 142.61 | 195.28 |
| 6 | 166.92 | 140.59 | 193.26 |
| 8 | 156.08 | 128.50 | 183.66 |
| 12 | 168.89 | 141.31 | 196.47 |
| 16 | 180.77 | 152.49 | 209.06 |
| 20 | 131.15 | 102.86 | 159.44 |
| 24 | 176.30 | 147.25 | 205.35 |
