# Supplementary material for: Conserved signatures of the canine faecal microbiome are associated with metronidazole treatment and recovery
Source: Sci Rep. 2024 Mar 4;14:5277. doi: 10.1038/s41598-024-51338-7 (PMC10912219; doi:10.1038/s41598-024-51338-7)
Supplement: Supplementary file 5 — Supplementary Table 3. [file 41598_2024_51338_MOESM5_ESM.docx]

### Table S3. Species Richness represented as observed ASV differences comparing mean differences (95% confidence intervals) between Week -1 (prior to treatment) and all other timepoints [Week 0 (treatment), Week 1 onwards (treatment cessation)]. *P*-values below 0.05 are considered statistically significant – denoted with an asterisk (*).

| Comparison | Mean difference | 95% lower | 95% upper | *P*-value |
| --- | --- | --- | --- | --- |
| 0 vs -1 | -103.74 | -136.37 | -71.11 | <1.00 x 10^-16^* |
| 0.5 vs -1 | -71.72 | -104.76 | -38.68 | 2.76 x 10^-8^* |
| 1 vs -1 | -12.60 | -45.63 | 20.44 | 0.93 |
| 2 vs -1 | 7.86 | -24.77 | 40.49 | 1.00 |
| 4 vs -1 | -20.34 | -53.39 | 12.71 | 0.49 |
| 6 vs -1 | -22.37 | -55.42 | 10.68 | 0.37 |
| 8 vs -1 | -33.21 | -67.19 | 0.78 | 0.06 |
| 12 vs -1 | -20.40 | -54.38 | 13.59 | 0.52 |
| 16 vs -1 | -8.52 | -43.04 | 26.01 | 1.00 |
| 20 vs -1 | -58.14 | -92.66 | -23.62 | 3.58 x 10^-5^* |
| 24 vs -1 | -12.99 | -48.09 | 22.11 | 0.94 |
