# Supplementary material for: Conserved signatures of the canine faecal microbiome are associated with metronidazole treatment and recovery
Source: Sci Rep. 2024 Mar 4;14:5277. doi: 10.1038/s41598-024-51338-7 (PMC10912219; doi:10.1038/s41598-024-51338-7)
Supplement: Supplementary file 6 — Supplementary Table 4. [file 41598_2024_51338_MOESM6_ESM.docx]

### Table S4. Faecal scores mean differences (95% confidence intervals) between Week -1 (prior to treatment) and all other timepoints [Week 0 (treatment), Week 1 onwards (treatment cessation)]. *P*-values below 0.05 are considered statistically significant – denoted with an asterisk (*).

| Comparison | Mean difference | 95% lower | 95% upper | *P*-value |
| --- | --- | --- | --- | --- |
| 0 vs -1 | 0.26 | -0.03 | 0.55 | 0.12 |
| 0.5 vs -1 | 0.07 | -0.23 | 0.36 | 1.00 |
| 1 vs -1 | 0.01 | -0.28 | 0.30 | 1.00 |
| 2 vs -1 | -0.13 | -0.41 | 0.16 | 0.86 |
| 4 vs -1 | -0.08 | -0.37 | 0.21 | 0.99 |
| 6 vs -1 | 0.09 | -0.21 | 0.38 | 0.99 |
| 8 vs -1 | -0.08 | -0.39 | 0.23 | 0.99 |
| 12 vs -1 | -0.13 | -0.43 | 0.17 | 0.87 |
| 16 vs -1 | -0.26 | -0.57 | 0.05 | 0.16 |
| 20 vs -1 | -0.16 | -0.47 | 0.14 | 0.65 |
| 24 vs -1 | -0.14 | -0.44 | 0.17 | 0.85 |
